# VIP/VPAC Axis Expression in Immune-Mediated Inflammatory Disorders: Associated miRNA Signatures

**DOI:** 10.3390/ijms23158578

**Published:** 2022-08-02

**Authors:** Amalia Lamana, David Castro-Vázquez, Hortensia de la Fuente, Ana Triguero-Martínez, Rebeca Martínez-Hernández, Marcelino Revenga, Raúl Villanueva-Romero, Mar Llamas-Velasco, Pablo Chicharro, Yasmina Juarranz, Mónica Marazuela, Marco Sales-Sanz, Rosario García-Vicuña, Eva Tomero, Isidoro González-Álvaro, Carmen Martínez, Rosa P. Gomariz

**Affiliations:** 1 Department of Cell Biology, Facultad de Biología y Facultad de Medicina, Universidad Complutense de Madrid, 28040 Madrid, Spain; amaliala@ucm.es (A.L.); dcastr01@ucm.es (D.C.-V.); ravillan@ucm.es (R.V.-R.); yashina@ucm.es (Y.J.); 2 Department of Immunology, Instituto de Investigación Princesa, Hospital Universitario de La Princesa, 28006 Madrid, Spain; hortensiadelafuente@gmail.com; 3 Department of Rheumatology, Instituto de Investigación Princesa Madrid, Hospital Universitario de La Princesa, 28006 Madrid, Spain; ana6n92@gmail.com (A.T.-M.); vicuna111@gmail.com (R.G.-V.); tomeroeva@yahoo.es (E.T.); isidoro.ga@ser.es (I.G.-Á.); 4 Department of Endocrinology, Instituto de Investigación Princesa, Hospital Universitario de La Princesa, 28006 Madrid, Spain; rbk_mar@yahoo.es (R.M.-H.); monica.hup@gmail.com (M.M.); 5 Department of Rheumatology, Hospital Universitario Ramón y Cajal, 28034 Madrid, Spain; marcelino.revenga@uah.es; 6 Department of Medicine and Medical Specialties, Universidad de Alcalá, 28805 Alcalá de Henares, Spain; 7 Department of Dermatology, Instituto de Investigación Princesa, Hospital Universitario de La Princesa, 28006 Madrid, Spain; mar.llamasvelasco@gmail.com (M.L.-V.); somniem@gmail.com (P.C.); 8 Department of Ophthalmology, Hospital Universitario Ramón y Cajal-IRYCIS, 28034 Madrid, Spain; salessanz@yahoo.es

**Keywords:** VIP, VPAC receptors, microRNAs, immune-mediated inflammatory disorders, rheumatoid arthritis, spondyloarthritis, Graves’ disease, psoriasis

## Abstract

Few studies have considered immune-mediated inflammatory disorders (IMID) together, which is necessary to adequately understand them given they share common mechanisms. Our goal was to investigate the expression of vasoactive intestinal peptide (VIP) and its receptors VPAC1 and VPAC2 in selected IMID, analyze the effect of biological therapies on them, and identify miRNA signatures associated with their expression. Serum VIP levels and mRNA of VPAC and miRNA expression in peripheral blood mononuclear cells were analyzed from 52 patients with psoriasis, rheumatoid arthritis, Graves’ disease, or spondyloarthritis and from 38 healthy subjects. IMID patients showed higher levels of VIP and increased expression of VPAC2 compared to controls (*p* < 0.0001 and *p* < 0.0192, respectively). Receiver operating characteristic curve analysis showed that the levels of VIP or VPAC2 expression were adequate discriminators capable of identifying IMID. Treatment of IMID patients with anti-TNFα and anti-IL12/23 significantly affected serum VIP levels. We identified miRNA signatures associated with levels of serum VIP and VPAC2 expression, which correlated with IMID diagnosis of the patients. The results indicate that the expression of VIP/VPAC2 is able of identify IMIDs and open up a line of research based on the association between the VIP/VPAC axis and miRNA signatures in immune-mediated diseases.

## 1. Introduction

Immune-mediated inflammatory disorders (IMIDs) include diseases that share an aberrant activation of immune response and chronic inflammation as the main pathogenic mechanism, and they affect around 3 to 7% of the population [1]. Patients with IMIDs present high morbidity, high degree of disability, and considerable reduction in quality of life [2].

The most prevalent IMIDs include rheumatoid arthritis (RA), spondyloarthritis (SpA), psoriasis (Ps), and Graves’ disease (GD), among others [2,3]. Given their heterogeneous nature, the management of these diseases represent significant diagnostic, therapeutic, and prognostic issues, and the search for appropriate biomarkers is currently a challenge for clinicians. Genome-wide association studies have demonstrated that genetic variants in loci conferring risk of developing autoimmune disorders are shared among IMIDs [4]. Therefore, epidemiological studies have suggested high contribution of nongenetic determinants and gene–environment interactions [5]. Moreover, it has been reported that about 50% of human genes are controlled by microRNAs (miRNAs). miRNAs are small, noncoding RNAs that are 18–25 nucleotides long and modulate protein expression at the post-transcriptional level [6]. Interaction of miRNA with a messenger RNA transcript can result in both gene silencing and gene activation being involved in the regulation of diverse physiological and pathological processes, including chronic inflammatory diseases. Thus, miRNAs regulate the expression of immune response genes, including genes of inflammatory cytokines, triggering loss of tolerance to self-antigens [7,8]. There has been increasing evidence about the presence of specific signatures of miRNAs that could be very useful as diagnosis and prognosis biomarkers for different pathologies, including autoimmune diseases [9,10,11].

Over the years, the potent anti-inflammatory effects and immunoregulatory capacity of vasoactive intestinal peptide (VIP), mediated by its receptors VPAC1 and VPAC2, have confirmed the potential of the VIP/VPAC axis in the management of various inflammatory and autoimmune disorders. Moreover, in the last decade, the VIP/receptor axis has emerged as a potential biomarker in autoimmune diseases. In this sense, decreased serum VIP levels have been described in a variety of disorders, including rheumatic diseases, such as early RA (eRA) [12], SpA [13], juvenile idiopathic arthritis [14], and osteoarthritis (OA) [15]; inflammatory bowel diseases, such as ulcerative colitis [16,17]; Graves’ disease [17]; Chagas cardiomyopathy [18]; Parkinson’s disease [19]; and asthma [20]. Regarding VIP receptors, it has been reported that VPAC1 and VPAC2 expression could reflect the clinical status in both eRA and GD [17,21].

Few studies have considered immune-mediated inflammatory disorders together, which is necessary for their accurate understanding. Thus, the main aim of the present study was to evaluate serum VIP levels and VPAC1 and VPAC2 expression in patients with different IMIDs and analyze the effect of biological therapies on the VIP/VPAC axis. In addition, we studied several miRNAs extracted from serum or peripheral blood mononuclear cells (PBMCs) that have been associated with the VIP/VPAC axis and other immunomodulatory molecules, such as IDO, CD69, STAT1, GAL1 and 9, or IGFR1. In the present study, we identified two miRNA signatures related to VIP/VPAC axis expression and IMID diagnosis in serum samples and PBMCs isolated from patients with different IMIDs.

## 2. Results

### 2.1. VIP: IMID Patients Show Higher Serum VIP Levels Than Controls

**Figure 1:**
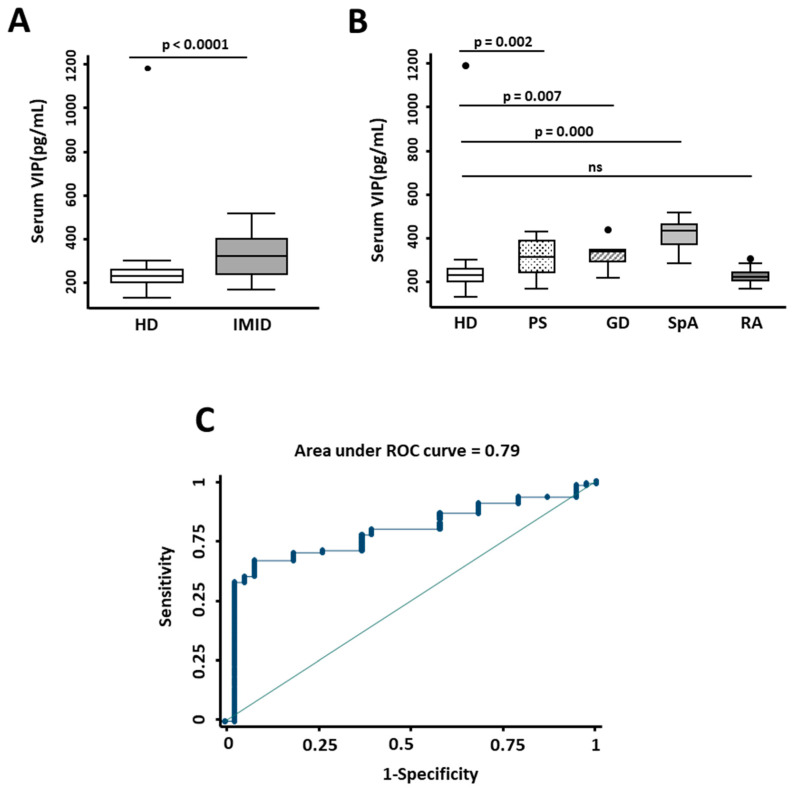
Expression of serum VIP levels in inflammatory/autoimmune pathologies. (**A**) Determination of serum VIP levels (pg/mL) by ELISA of 38 healthy donors (HD) and 52 patients with immune-mediated inflammatory diseases (IMID). Statistical significance was determined using the variable of serum VIP levels normalized by inverse square root and Student’s *t*-test to obtain *p*-value < 0.0001. (**B**) Serum VIP levels (pg/mL) of 38 healthy donors (HD) and 15 patients with psoriasis (PS), 8 with Graves’ disease (GD), 15 with spondyloarthritis (SpA), and 14 with rheumatoid arthritis (RA) are shown. Statistical significance was calculated using the variable of serum VIP levels normalized by inverse square root and applying ANOVA and Bonferroni correction for multiple comparisons to obtain *p*-values as indicated. In all panels, data are presented as the interquartile range (p75: upper edge of the box, p25: bottom edge of the box, and p50: midline) and p90 and p10 (lines below and above the box) of the serum VIP levels. Dots represent outliers. Significance threshold was set at *p* < 0.05. (**C**) The graph shows the receiver operating characteristic (ROC) curve analysis to assess the ability of serum VIP levels to discriminate between patients with IMID and healthy donors. Area under ROC curve is indicated in the graph.

In order to determine whether there were differences in the VIP/VPAC axis between IMID patients and the control group, we first analyzed the expression of VIP in serum. The clinical characteristics of these patients are explained in the Materials and Methods section and shown in Table 1. As shown in Figure 1A, serum VIP levels in IMID patients were significantly higher than in the control group. Moreover, ROC curve analysis suggested that serum VIP concentration above 216 pg/mL could discriminate IMID patients from controls (AUC = 0.79) with 70.25% sensitivity and 81.58% specificity (LR+ 3.81 and LR− 0.364; Figure 1C). On the other hand, when we analyzed serum VIP levels in different pathologies, we observed that they were significantly higher in psoriasis (*p* = 0.002), Graves’ disease (*p* = 0.007), and spondyloarthritis (*p* ≤ 0.0001), compared to controls, whereas RA patients showed similar VIP levels to healthy donors (Figure 1B).

### 2.2. Receptors: VPAC2 Expression Is Higher in IMID Patients Than in Controls

**Figure 2:**
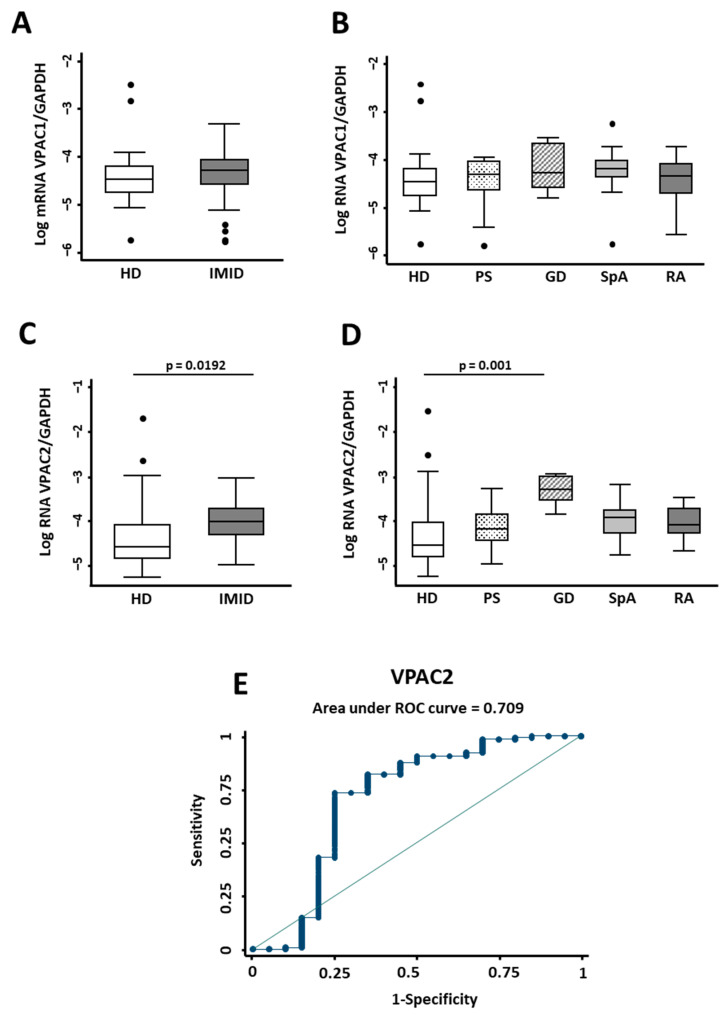
Expression of the VIP/receptor axis in inflammatory/autoimmune pathologies. (**A**) The relative expression of *VPAC1*/*GAPDH* mRNA normalized by log transformation of 38 healthy donors (HD) and 52 patients with immune-mediated inflammatory diseases (IMID) is shown. Statistical significance was determined with Student’s *t*-test. No significant differences were observed. (**B**) Log of relative expression of *VPAC1*/*GAPDH* mRNA of 38 healthy donors (HD) and 15 patients with psoriasis (PS), 8 with Graves’ disease (GD), 15 with spondyloarthritis (SpA), and 14 with rheumatoid arthritis (RA) is shown. (**C**) Log of relative expression of *VPAC2*/*GAPDH* mRNA of 38 healthy donors (HD) and 52 patients with immune-mediated inflammatory diseases (IMID) is shown. Statistical significance was calculated using relative expression of *VPAC2*/*GAPDH* mRNA normalized by inverse square root using Student’s *t*-test. The *p*-value is shown in the figure. (**D**) Log of relative expression of *VPAC2*/*GAPDH* mRNA of 38 healthy donors (HD) and 15 patients with psoriasis (PS), 8 with Graves’ disease (GD), 15 with spondyloarthritis (SPA), and 14 with rheumatoid arthritis (RA) is shown. Statistical significance was calculated using inverse square root of relative expression of VPAC2/GAPDH mRNA by applying the ANOVA test and Bonferroni correction for multiple comparisons. Significant differences between groups are shown in the figure. In all panels, data are presented as the interquartile range (p75: upper edge of the box, p25: bottom edge of the box, and p50: midline) and p90 and p10 (lines below and above the box) of the serum VIP levels. Dots represent outliers. Significance threshold was set at *p* < 0.05. (**E**) The graph shows the ROC curve analysis to assess the ability of relative expression of VPAC2/GAPDH to discriminate between patients with IMID and healthy donors. Area under ROC curve is indicated in the graph.

VPAC1 receptor transcript analysis showed no differences between IMID patients and the control group for IMID in general or for each specific disease (Figure 2A,B). However, we observed that *VPAC2* expression in IMID patients was significantly higher than in healthy donors (*p* = 0.0192) (Figure 2C). Nevertheless, when we analyzed each pathology individually, only Graves’ disease exhibited significant differences compared to the control group (*p* = 0.001) (Figure 2D). ROC curve analysis demonstrated that 2^−ΔCt^ *VPAC2*/*GAPDH* mRNA expression above 0.0154 could classify IMID patients from controls (AUC = 0.7) with 73.44% sensitivity and 75.00% specificity (LR+ 2.94 and LR− 0.3542; Figure 2E).

### 2.3. Biological Therapies in IMID Patients Affect Serum VIP Levels

**Figure 3:**
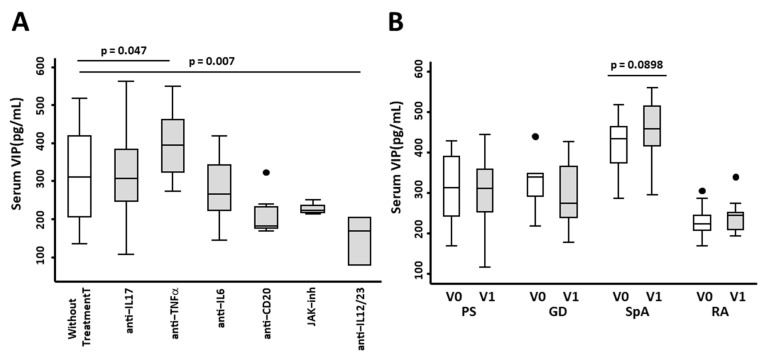
Effect of biological therapies on the expression of serum VIP levels in patients with immune-mediated inflammatory diseases (IMIDs). (**A**) Serum VIP levels (pg/mL) of 38 healthy donors without treatment and 9 IMID patients with anti-IL17 treatment, 15 with anti-TNFα treatment, 10 with anti-IL6 treatment, 8 with anti-CD20 treatment, 4 with JAK inhibitors, and 2 with anti-IL12/23 treatment are shown. Statistical significance was calculated using the variable of serum VIP levels normalized by inverse square root and applying ANOVA and Bonferroni correction for multiple comparisons to obtain the *p*-values as indicated. In all panels, data are presented as the interquartile range (p75: upper edge of the box, p25: bottom edge of the box, and p50: midline) and p90 and p10 (lines below and above the box) of the serum VIP levels. Dots represent outliers. Significance threshold was set at *p* < 0.05. (**B**) Serum VIP levels (pg/mL) by ELISA of 15 patients with psoriasis (PS), 8 with Graves’ disease (GD), 15 with spondyloarthritis (SpA), and 14 with rheumatoid arthritis (RA) before (V0) and after 2–4 months (V1) of treatment with biological therapies are shown. To analyze the differences in serum VIP levels before and after treatment, paired sign test was used. Significant differences between groups are shown in the figure.

To determine whether therapy with biological treatment influences serum VIP levels, we first studied whether different biological therapies (Table 2) were associated with changes in serum VIP levels. Our data showed that treatment with anti-TNFα raised serum VIP levels (*p* = 0.047) (Figure 3A), whereas treatment with anti-IL12/23 decreased circulating VIP (Figure 3A). Next, we carried out a pairwise analysis between nontreated patients and patients after two and four months of treatment. We reported no differences in PS, GD, and RA. Moreover, in SpA, we observed higher levels of serum VIP after treatment with biological therapies (*p* = 0.089) (Figure 3B).

VPAC1 and VPAC2 gene expression showed no difference in patients treated with biological therapies (Figure S1).

### 2.4. Identification of miRNA Signatures Associated with Serum Levels of VIP in IMID Patients

Given the crucial role of miRNAs in a variety of autoimmune and chronic inflammatory diseases targeting many immunoregulatory molecule genes, we evaluated the serum expression of a panel of miRNAs related to immune response and their association with serum levels of VIP.

**Figure 4:**
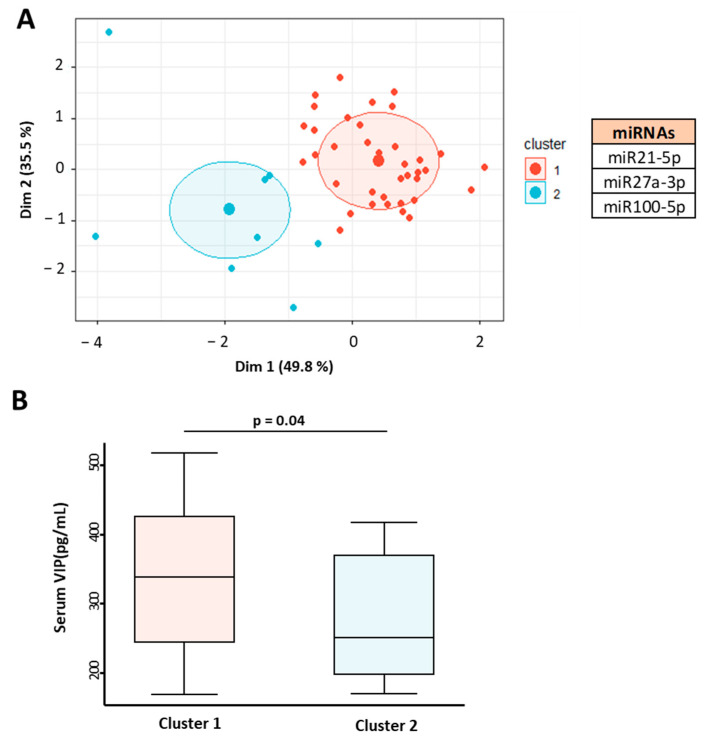
Association of miRNA profile in patients with immune-mediated inflammatory diseases (IMID) with serum levels of VIP. (**A**) Graphical representation of cluster analysis based on miR215p, miR27a-5p, and miR100-5p expression in 45 IMID patients. K-means clustering based on Euclidean distance and principal component analysis (PCA) was applied. (**B**) Serum VIP levels (pg/mL) of IMID patients according to their association with miRNA expression cluster. Statistical significance was calculated using the variable of serum VIP levels normalized by inverse square root and applying Student’s *t*-test. Data are presented as the interquartile range (p75: upper edge of the box, p25: bottom edge of the box, and p50: midline) and p90 and p10 (lines below and above the box) of the serum VIP levels. Significance threshold was set at *p* < 0.05.

Consistent clustering analysis of IMID patients was performed based on miRNA expression associated with serum VIP levels (miR21-5p, miR27a-3p, and miR100-5p). Principal component analysis showed two miRNA expression clusters in patients with no overlapping area between them (Figure 4A).

When comparing the serum levels of VIP in the two groups, significant differences were observed (*p* = 0.04) (Figure 4B). Cluster 1 was associated with high levels of VIP and cluster 2 with lower levels, showing that this miRNA profile is capable of distinguishing patients by their serum VIP concentration.

### 2.5. miRNA Profile Is Associated with VPAC Expression in IMID

Next, we performed K-means clustering analysis and cluster analysis to evaluate miRNA expression in PBMCs from IMID patients based on their role in the immune response, dysregulated expression in autoimmune diseases, or predictive binding to VPAC mRNA.

**Figure 5:**
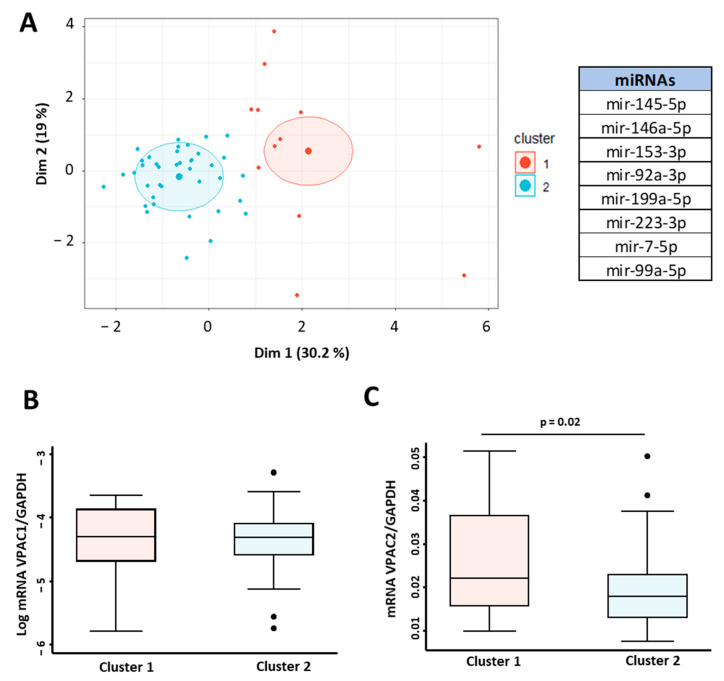
miRNA signature associated with VPAC receptors gene expression. (**A**) Cluster analysis in 52 IMID patients based on the expression of miRNAs miR145-5p, miR146A-5p, miR153-3p, miRNA92a-3p, miR199a-5p, miR223-3p, miR7-5p, and miR99a-5p. K-means clustering based on Euclidean distance and PCA was used for graphical representation. Log of relative expression (2^−ΔCt^) of *VPAC1*/*GAPDH* (**B**) and *VPAC2*/*GAPDH* mRNA (**C**) according to their association with miRNA expression cluster. Statistical significance was calculated using Student’s *t*-test. For both datasets, data are presented as the interquartile range (p75: upper edge of the box, p25: bottom edge of the box, and p50: midline) and p90 and p10 (lines below and above the box) of the relative gene expression. Dots represent outliers. Significance threshold was set at *p* < 0.05.

First, we assessed the correlations between expressions of *VPAC1* and *VPAC2* and miRNAs (miR145-5p, miR146A-5p, miR153-3p, miRNA92a-3p, miR199a-5p, miR223-3p, miR7-5p, and miR99a-5p). Two clusters were retained using the K-means clustering analysis to characterize the IMID population (Figure 5A).

No differences in the expression of VPAC1 were found among the two clusters (Figure 5B). On the contrary, the differences in expression of *VPAC2* were statistically significant between the two groups (*p* = 0.02) (Figure 5C).

### 2.6. Predictive Value of miRNA Signatures in IMID Diagnosis

To confirm the value of VIP and VPAC2 expression in the diagnosis of IMID, we examined whether the identified clusters could discriminate between pathologies.

The results are presented in Table 3 and Table 4. As expected, the cluster associated with high serum levels of VIP was able to confirm the diagnosis of all pathologies with the exception of rheumatoid arthritis (Pearson χ^2^ = 6.7218, Pr = 0.081, Table 3).

Interestingly, the analysis revealed significant differences in diagnosis across the two clusters associated with *VPAC2* expression (*p* < 0.023) (Pearson χ^2^ = 9.5731, Pr = 0.023, Table 4), highlighting the relevance of the increase in VPAC2 expression in IMIDs.

## 3. Discussion

Immune-mediated inflammatory disorders are a group of pathologies where the common feature is inflammation. They affect 15% of the population and represent a heavy socioeconomic burden. Despite increasing knowledge about the etiopathogenesis and marked progress in their management, there is a lack of diagnostic markers. Previous evidence supports the VIP/receptors axis as a prognostic and diagnostic marker for various inflammatory/autoimmune diseases. In the current study, we analyzed serum VIP levels and the expression of VPAC1 and VPAC2 in different IMIDs, including PS, GD, SpA, and RA. The results pointed out the uniqueness of each disease but showed a common signature for these pathologies as a consequence of the common mechanisms they share. We found that a serum VIP concentration above 216 pg/mL could differentiate IMID patients from controls. Analyzing each pathology individually, we observed that in PS, GD, and SpA, VIP levels were significantly higher than controls, whereas the values in RA were similar to those observed in controls.

In the literature, the information provided to date on circulating VIP levels in different inflammatory/autoimmune diseases is variable. Previous studies have shown reduced levels of serum VIP in GD at the onset of the disease in the hyperthyroid status and no significant variations in the normal and hypothyroid status.

Regarding SpA, we earlier found no differences in serum VIP levels between SpA and patients with nonspecific lower back pain. Moreover, we have reported the association between low serum VIP concentration and increased disease severity in patients with early SpA, namely reduced functional status, bone edema in MRI, and a more intense inflammatory burden (anemia, psoriasis, inflammatory bowel disease, and enthesitis). Moreover, another study described higher levels of serum VIP in ankylosing spondylitis patients compared to healthy donors and reported a significant association with platelet count [22].

Previous findings in psoriasis appear more homogeneous and point to higher serum VIP levels in patients with psoriasis. Elevated expression of VIP in the plaques and plasma of psoriasis patients have been reported [23,24]. However, a recent study observed no difference in serum VIP levels between controls and patients [25].

Regarding RA, our current results corroborate that patients with established RA have serum VIP expression levels similar to the control group, as had been observed in patients with recent-onset arthritis [12]. However, in early arthritis, we observed that those patients with lower levels had worse prognosis at 2-year follow-up, suggesting that the VIP signaling has a relevant role in the control of the pathology.

All in all, the current results indicate that high serum VIP levels with respect to controls are adequate discriminators capable of identifying IMIDs. However, the results are in contrast with a plethora of previous nonhomogeneous results for different pathologies. Possible explanations for this divergence include the difficulty in obtaining homogeneous groups in terms of age, sex, or population and early versus long-term disease stage. The heterogeneity of the stage of the disease can be a determining factor as patients may be in early stages, i.e., without treatment, or in stages in which the patient has already begun to receive therapy. In this sense, our IMIDs patients represent a homogeneous population with an established diagnosis who have received previous treatments and are finally receiving biologic therapy.

Therefore, one of the hypotheses that would explain the discrepancy in the elevated levels in this type of patients is that the specific treatment would be able to increase the levels of VIP that would be protective. In this regard, our data showed that treatment with anti-TNFα, the first approach for biological treatment of these diseases after failure of therapy with FAMES, raised serum VIP levels. These data support previous findings that treatment with anti-TNFα agents increase serum VIP levels in early arthritis and in SpA [12,13]. Moreover, we have reported cases of early arthritis where combinations of genetic variants of VIP gene were associated with treatment requirements. Thus, the genotype associated with high VIP levels is also related to less severe disease and thus to reduced need for intensive treatment [26]. When we analyzed the effect of different biological therapies in patients with IMIDs, we observed a significant increase in serum VIP levels in SPA patients who mainly received anti-TNFα. These data suggest that the most effective biological drug for positive regulation of VIP levels is anti-TNFα. More studies are needed to strongly test this hypothesis with a larger number of patients in the remaining pathologies studied. A recently published study corroborates this finding in COVID-19 patients, where the plasma VIP levels were raised in patients with severe COVID-19, correlating with reduced inflammatory mediators and with survival in these patients [27].

The main signaling pathway of VPAC receptors is their coupling to G-proteins that catalyze cAMP synthesis and also mediate the increase in Ca^P^. The VIP/receptor axis inhibits AP-1 and IRF activation through a PKA-dependent mechanism and can also prevent NFκB translocation to the nucleus, impeding IKK activation through a cAMP-independent mechanism and thus interfering in crucial signaling inflammatory pathways [28,29]. Both receptors exert the same mechanisms in the control of immune response. However, different studies have shown increased functionality of VPAC2 in both cellular activation and pathological conditions.

When we analyzed the expression of receptors, we observed that there were no differences in the expression of *VPAC1* between IMID patients and control group for IMID in general or for each specific disease. However, *VPAC2* expression allowed us to establish a cut-off point to discriminate between IMID pathologies. Specifically, a value 0.0154 of 2^−ΔCt^ *VPAC2*/*GAPDH* of mRNA expression could classify IMID patients from controls. VPAC2 expression was found to be increased in all IMID pathologies studied, reaching significance in patients with GD as well as in IMID as a whole.

The importance of VPAC receptor expression in PBMCs has been previously evaluated in only two of the IMID pathologies studied: early arthritis and GD. In GD, higher expression of both VPAC receptors was found. However, VPAC1 signaling was impaired, whereas a significant increase in VPAC2 expression was characterized as a mechanism that helps balance VPAC1 dysfunction by upregulating expression of the functional receptor [17,28].

In our study, *VPAC1* expression levels in RA did not differ from controls, which is in agreement with data previously described for early arthritis [21].

However, *VPAC2* gene expression in PBMCs isolated at baseline visit from early arthritis patients has been described as being significantly higher than that of controls [21]. In the current study, in patients with established RA and active pathology, a trend towards higher levels of *VPAC2* expression was corroborated. These data are in agreement with the increased expression of VPAC2 in synovial fibroblasts observed in RA patients, which is related to its anti-inflammatory action [30]. The higher expression of VPAC2 receptor could be interpreted as a reinforcement and compensation mechanism when there is loss of VPAC1 receptor function under pathological conditions. In this sense, VPAC2 has also been shown to be the predominant receptor in PBMC in early arthritis in different experimental conditions, such as activated memory Th cells, Th17-polarized cells, and proinflammatory CD4+CD28− T cells [31,32,33]. Moreover, this increased expression has been described in monocytes from Sjögren syndrome [34] and CD4 T cells from multiple sclerosis [35]. It should also be noted that none of the biological therapies affected the expression of receptors.

A major strength of our data resides on the fact that we have searched for markers that may not only be specific for each disease but may also expose shared events present in several IMIDs, thereby opening up prevailing, although not yet understood, relationships between different types of IMIDs. Thus, we have reported that certain values of circulating VIP levels and gene expression of one of the receptors, VPAC2, can differentiate IMID patients from healthy patients.

MicroRNAs (miRNAs) play a crucial role in immune system function and immune tolerance, and altered expression of miRNAs and their target genes have been shown to contribute to the pathophysiology of many immune disorders [8,36].

To date, this is the first study to report the relationship between miRNA profiles and VIP and their receptors in autoimmune diseases. In fact, studies on the association or regulation of VIP/VPAC by miRNA are very scarce.

The first reported study showed that upregulation of miR-525-5p in LPS-activated peripheral blood monocytes reduced *VPAC1* expression [37]. Afterwards, a study on rat RT4 Schwann cell line exposed to LPS described a subset of deregulated miRNAs (including miR-21 and miR-146) that correlated with the expression of VIP axis [38]. In this sense, we earlier demonstrated the association between serum VIP levels and an allelic variant in VIP gene in patients with early arthritis through a miRNA-mediated mechanism [26].

In the present study, we analyzed the association between immunologically relevant miRNAs, both in PBMCs and serum, and expression of the VIP/VPAC axis in IMID. We identified two miRNA profiles, a signature capable of distinguishing IMID patients by their serum VIP concentrations and a distinctive miRNA profile associated with *VPAC2* mRNA expression levels in PBMCs.

Here, we found an association between the circulating miRNAs miR-21, miR-27, and miR-100 and serum levels of VIP. In agreement with our data, miR-21 has largely been associated with the development of autoimmune diseases, playing an essential role in the regulation of the immune system [36,39]. Moreover, miR-27 functions as a key regulator in Treg development and function, and a tight regulation of miR-27 has been identified as essential to maintain Treg-mediated immune tolerance and regulate Th2 differentiation and function [40]. Moreover, altered expression and editing of miRNA-100 affects Treg differentiation [41].

We found a second miRNA profile associated with *VPAC2* mRNA expression levels in IMID patients (miR145-5p, miR146a-5p, miR153-3p, miRNA92a-3p, miR199a-5p, miR223-3p, miR7-5p, and miR99a-5p).

Circulating miR-145 has recently been described as a marker of therapeutic response to anti-TNF in patients with ankylosing spondylitis [42]. Moreover, this miRNA negatively regulates osteogenesis and chondrogenesis [43,44], meaning it could be of potential diagnostic value in RA [45].

Regarding miR-146a, its overexpression in serum of active ankylosing spondylitis patients suggests that it could be used as a diagnostic biomarker [46]. It has also been reported that this miRNA is upregulated in AR and is essential for the control of pathogenic Th1 responses by Treg cells [47].

With respect to miR-153, it has been suggested that it may be a promising therapeutic target for the treatment of inflammation-associated diabetes [48].

The involvement of miR-92a in inflammatory processes, including systemic lupus erythematosus (SLE) and scleroderma, has been outlined [49,50], along with its involvement in the pathogenesis of experimental autoimmune encephalitis, probably as a positive regulator of pathogenic Th1 differentiation [51].

miR-99a promotes the differentiation of Treg cells [52], and miR-199 is involved in the development of Th17 and Treg cells in patients with multiple sclerosis [53], showing increased expression in some autoimmune diseases, such as atopic eczema and SLE [54]. Moreover, the expression of miR-7 was found to be increased during the activation of TLR4 signaling, suggesting that it could act as a new negative fine-tuner in the regulation of its signaling [55].

Lastly, miR-223 is dysregulated in many inflammation-related disorders. It is overexpressed in the synovium and peripheral T cells of patients with RA [56,57] and has emerged as a potential predictive biomarker of RA risk [58].

In contrast to VPAC2, we did not find an association between the studied miRNAs and the expression levels of VPAC1 in IMID patients. Numerous studies have demonstrated that VPAC1 is constitutively expressed at higher levels than VPAC2 in resting human monocytes/macrophages and lymphocytes, while VPAC2 expression is induced by immune stimulation [28,59]. Furthermore, we have previously demonstrated that a more severe inflammation, seen as high levels of IL-6, is associated with lower expression of VPAC1 in PBMCs from patients with early arthritis and, conversely, with increased expression of VPAC2 [21]. Given that miRNAs are essential for adequate control of expression of genes in autoimmune diseases, taken together, these data could suggest greater impact of epigenetic regulators, such as miRNA, on *VPAC2* expression. However, more research is required to explain the dynamic regulation of these receptors in inflammation and autoimmunity.

A limitation of this study is that our data are descriptive, and we cannot determine the causality of the association between miRNAs and the VIP/VAPAC axis in IMID patients. In silico prediction (TargetScan 8.0 program, https://www.targetscan.org and miRbase, www.mirbase.org; accessed on 7 May 2022) revealed possible binding sites of three miRNAs to VIP or VPAC mRNA, but these were poorly conserved (miR-27 to VIP and VPAC2, miR-199 to VPAC1 and VPAC2, and miR-7 to VIP). We do not know whether the analyzed miRNAs could exert a direct or indirect effect on the expression of the axis. Functional studies would be necessary to elucidate this finding, but it opens a line of research that could be explored.

Interestingly, the miRNA signatures that distinguish patients by their serum VIP levels could identify different IMIDs, except rheumatoid arthritis, confirming the diagnostic value of VIP concentration. In the same way, we demonstrated the predictive value of miRNA signatures associated with *VPAC2* expression levels in IMID diagnosis.

Most of the studies on the association between miRNAs and immune-mediated disorders have focused on a particular pathology, and there are barely any data on a set of pathologies [60]. Currently, patients suffering from systemic autoimmune diseases present complex diagnostic, therapeutic, and prognostic problems in daily clinical practice, and some patients may even have more than one autoimmune disorder at the same time. Although our results need validation in larger patient cohorts, they show the potential of the VIP/VPAC axis in the diagnosis of IMID and open a line of research based on its association with miRNA signatures in autoimmune diseases, providing an innovative approach in the management of these disorders as a whole given their shared common mechanisms.

## 4. Materials and Methods

### 4.1. Patients and Samples

The samples used in this work were obtained in the context of the BIOIMID project, which was approved by the Ethics Committee for Clinical Drug Research of Hospital de La Princesa (CEIM Hospital Universitario La Princesa, PI-734).

For the present study, samples from a total of 90 subjects were analyzed: 38 healthy donors (HD), 15 patients with psoriasis (PS), 8 with Graves’ disease with ophthalmopathy (GD) defined by the EUGOGO criteria [61], 15 with spondyloarthritis (SpA) (8 fulfilled the New York classification criteria for ankylosing spondylitis [62], 2 had undifferentiated spondylitis fulfilled the Assessment of SpondyloArthritis International Society (ASAS) criteria for SpA [63], and 2 had psoriatic arthritis (PsA) who fulfilled the Classification Criteria for Psoriatic Arthritis (CASPAR) [64]), and 14 patients classified as having rheumatoid arthritis (RA) according to the 2010 EULAR/ACR criteria [65] who all fulfilled the 1987 diagnosis criteria for RA [66]. Written informed consent was obtained from all of them prior to their inclusion in accordance with the Declaration of Helsinki. The main clinical characteristics of patients and controls are shown in Table 1 and Table 2.

All patients received treatment with biological drugs as shown in Table 2. Blood samples were taken before and after (in a period of 2–4 months) treatment prescription. Serum was obtained by centrifugation (1500× *g*) for 10 min from 10 mL of whole blood and stored at −80 °C until use in the Instituto de Investigación Sanitaria La Princesa (IIS-IP) Biobank for translational research.

Response to treatment was classified according to the criteria for each pathology. In patients with PS, the Psoriasis Area and Severity Index (PASI) was used [67], which classifies patients with PASI 90 as responders, PASI 75–50 as intermediate responders, and PASI below 50 as nonresponders [68]. In RA and PsA patients, response to treatment was defined according to the EULAR criteria [69]. In patients with SpA and AS, BASDAI was used [70], where patients with BASDAI reduction of 50% is classified as responders, 20–50% as intermediate responders, and below 20% as nonresponders, as indicated by Rudwaleit M et al. [71]. In the case of patients with GD, response to treatment was assessed as improvement in ophthalmopathy.

### 4.2. Measurement of Serum VIP Levels

Serum VIP levels were measured using a commercial enzyme immunoassay kit (Phoenix Pharmaceuticals, Karlsruhe, Germany) following the manufacturer’s instructions. A total of 142 serum samples (38 from the control group and 104 from IMIDs) were analyzed before and after treatment. To avoid interassay variability of this test, all samples corresponding to consecutive visits from the same patient were measured together on the same plate, and patients with different IMID pathologies were included in each plate.

### 4.3. RNA Extraction and qRT-PCR

To analyze mRNA levels of the VIP receptors (*VPAC1* and *VPAC2*), RNA was extracted from peripheral blood mononuclear cells (PBMCs) using TRIsure™ (Bioline, Luckenwalde, Germany) and retrotranscribed to cDNA using a high-capacity cDNA reverse transcription kit (Thermo Fisher, Madrid, Spain) following the manufacturer’s instructions. Subsequently, the expression of *VPAC1* and *VPAC2* mRNA was analyzed by quantitative real-time PCR (qRT-PCR). The endogenous expression of glyceraldehyde-3-phosphate dehydrogenase (*GAPDH*) was normalized using the formula 2^−ΔCt^. The qRT-PCR reaction was carried out using real-time ready assay probes (Assay ID 104081, 148318, and 141139 for *VPAC1*, *VPAC2*, and *GAPDH,* respectively; Roche Life Science, Barcelona, Spain) and LightCycler^®^ 480 SYBR Green I Master according to the manufacturer’s instructions. Amplification was performed on a LightCycler^®^ 480 Instrument II (Roche Life Science).

### 4.4. miRNA Expression

To analyze the expression of miRNAs, total RNA was purified from 200 μL of serum samples using miRNeasy Serum/Plasma Advanced kit (Qiagen, Hilden, Germany) according to the manufacturer’s instructions. Before RNA isolation, the absorbance of free hemoglobin at 414 nm was measured using a NanoDrop, and samples with value >0.2 were discarded. RNA spike-in templates UniSp2, UniSp4, and UniSp5 were added to 200 μL of serum to control the isolation step. RNA was purified on microRNA mini spin column and stored at −80 °C.

For PBMC samples, total RNA was isolated using the miRNAeasy Micro kit (Qiagen) following the manufacturer’s instructions.

Reverse transcription (RT) was performed with 2 μL of RNA obtained from serum samples or 10 ng of RNA obtained from PBMC samples for a final reaction of 10 μL using the miRCURY LNA RT kit (Qiagen).

To perform qRT-PCR, cDNA samples obtained from serum were diluted 1:30 with nuclease-free water, while those obtained from PBMCs were diluted 1:80. The amplification reaction was performed in triplicate with microRNA LNA™ PCR primer sets (Qiagen) in 384-well customized plates (miRCURY LNA miRNA Custom PCR Panels, Qiagen) in a CFX384 Real-Time System (Roche). Plates also included primers as interplate calibration (IPC) for UniSp3 and as control RT for UniSp6. For cDNA from serum samples, plates also included UniSp2 as RNA extraction control.

### 4.5. qPCR Data Analysis

CT values above cycle 37 were removed. Data were normalized using the geometric mean of miR-23a, miR-484, miR-23b, miR-24-3p, and miR-15b-5p for serum samples and miR-22-3p and miR-326 for PBMC samples. These miRNAs were selected as the more stable miRNAs using the software packages Normfinder and geNorm.

### 4.6. Statistical Analysis

Descriptive results were expressed as median and interquartile range (IQR). Some variables did not show a Gaussian distribution, so data were normalized. Serum VIP levels and relative *VPAC2* gene expression were normalized through inverse square root, and relative *VPAC1* gene expression was normalized through logarithmic transformation. Statistical analyses were performed using Stata 16 for Windows (Stata Corp LP, College Station, TX, USA) and R (4.1.2 Version).

Differences between groups were compared using Student’s *t*-test or analysis of variance (ANOVA) as appropriate. To analyze differences in VIP expression in patients before and after treatment, we used the nonparametric sign test.

Receiver operating characteristic (ROC) curve analysis (roctab command option *graph* of Stata) was used to study the ability to discriminate between healthy donors and IMID patients from serum VIP levels or normalized VPAC2/GAPDH mRNA expression levels using the formula 2^−ΔCt^. We selected cut-off points based on the best trade-off values between sensitivity, specificity, correctly classified cases, and positive (LR+) and negative (LR−) likelihood ratios obtained using the roctab detail command.

Spearman’s bivariate correlations were performed between serum VIP levels and VPAC1 and VPAC2 mRNA expression and all miRNAs studied in order to choose miRNAs to be included in subsequent analyses.

For the determination of miRNA signature, only patients with no missing data were included due to the requirements of further analysis. The Factoextra R package (https://CRAN.R-project.org/package=factoextra; accessed on 20 January 2022) was used to perform clustering based on Euclidean distance, and principal component analysis (PCA) was applied to show the clustering of samples with the first two components. For representation of graphics, the package *ggpubr* was used (https://CRAN.R-project.org/package=ggpubr; accessed on 20 January 2022).

## Figures and Tables

**Table 1:** Clinical features of patients.

	HD	PS	GD	SpA	RA
**n**	38	15	8	15	14
**Gender (Female)**	26	9	8	7	10
**Age (years)**	48.6 (33.8–62.5)	37 (31.1–50.8)	57.8 (57.1–58.4)	45.3 (28.3–51.7)	60.7 (47.2–74.9)
**Disease Duration (months)**		5.5 (3.4–6.9)	20 (18–24)	74.9 (40–125.8)	75.3 (32.9–176.7)
**Response to treatment (%)**					
No responder		5.2	28	6.9	7.1
Intermediate responder		5.3	28	20.7	28.6
Responder		89.5	44	72.4	64.2

Values show number for categorical values and median (25–75 interquartile intervals) for continuous variables. HD: healthy donors; PS: psoriasis; GD: Graves´ disease; SpA: pondyloarthritis; RA: rheumatoid arthritis.

**Table 2:** Treatment received by patients with immune-mediated inflammatory disorders (IMID).

Treatment	PS	GD	SpA	RA	Total
Secukinumab (anti-IL17)	4		3		7
Ixekizumab (anti-IL17)	2				2
Adalimumab (anti-TNFα)	4		4		8
Certolizumab (anti-TNFα)			7		7
Ustekinumab (anti-IL12/23)	2				2
Rituximab (anti-CD20)				8	8
Tocilizumab (anti-IL6)		8		2	10
Baricitinib (JAKs inhibitors)				4	4

PS: psoriasis; GD: Graves´ disease; SpA: spondyloarthritis; RA: rheumatoid arthritis.

**Table 3:** miRNA clusters associated with elevated serum VIP levels are useful in determining the diagnosis.

	Cluster 1	Cluster 2	Total
**PS**	14	2	16
**GD**	3	0	3
**SpA**	13	1	14
**RA**	7	5	12
**Total**	37	8	45

PS: psoriasis; GD: Graves´ disease; SpA: spondyloarthritis; RA: rheumatoid arthritis.

**Table 4:** miRNA clusters associated to *VPAC2* expression are useful in determining the diagnosis.

	Cluster 1	Cluster 2	Total
**PS**	4	12	16
**GD**	5	3	8
**SpA**	2	13	15
**RA**	1	12	13
**Total**	12	40	52

PS: psoriasis; GD: Graves´ disease; SpA: spondyloarthritis; RA: rheumatoid arthritis.

## Data Availability

All relevant data are within the paper and its Supporting Information files. The datasets generated and/or analyzed during the current study are not publicly available due to the confidential nature of the clinical data but are available from the corresponding author upon reasonable request.

## Supplementary Materials

The supporting information can be downloaded at: https://www.mdpi.com/article/10.3390/ijms23158578/s1.
