# Supplementary material for: VIP/VPAC Axis Expression in Immune-Mediated Inflammatory Disorders: Associated miRNA Signatures
Source: Int J Mol Sci. 2022 Aug 2;23(15):8578. doi: 10.3390/ijms23158578 (PMC9369218; doi:10.3390/ijms23158578)
Supplement: Supplementary file 1 [file ijms-23-08578-s001.zip › ijms-1830260-supplementary.pdf]

Supplementary material

Figure S1. Effect of biological therapies treatment in patients with IMIDs on the expression of the VIP–receptors.

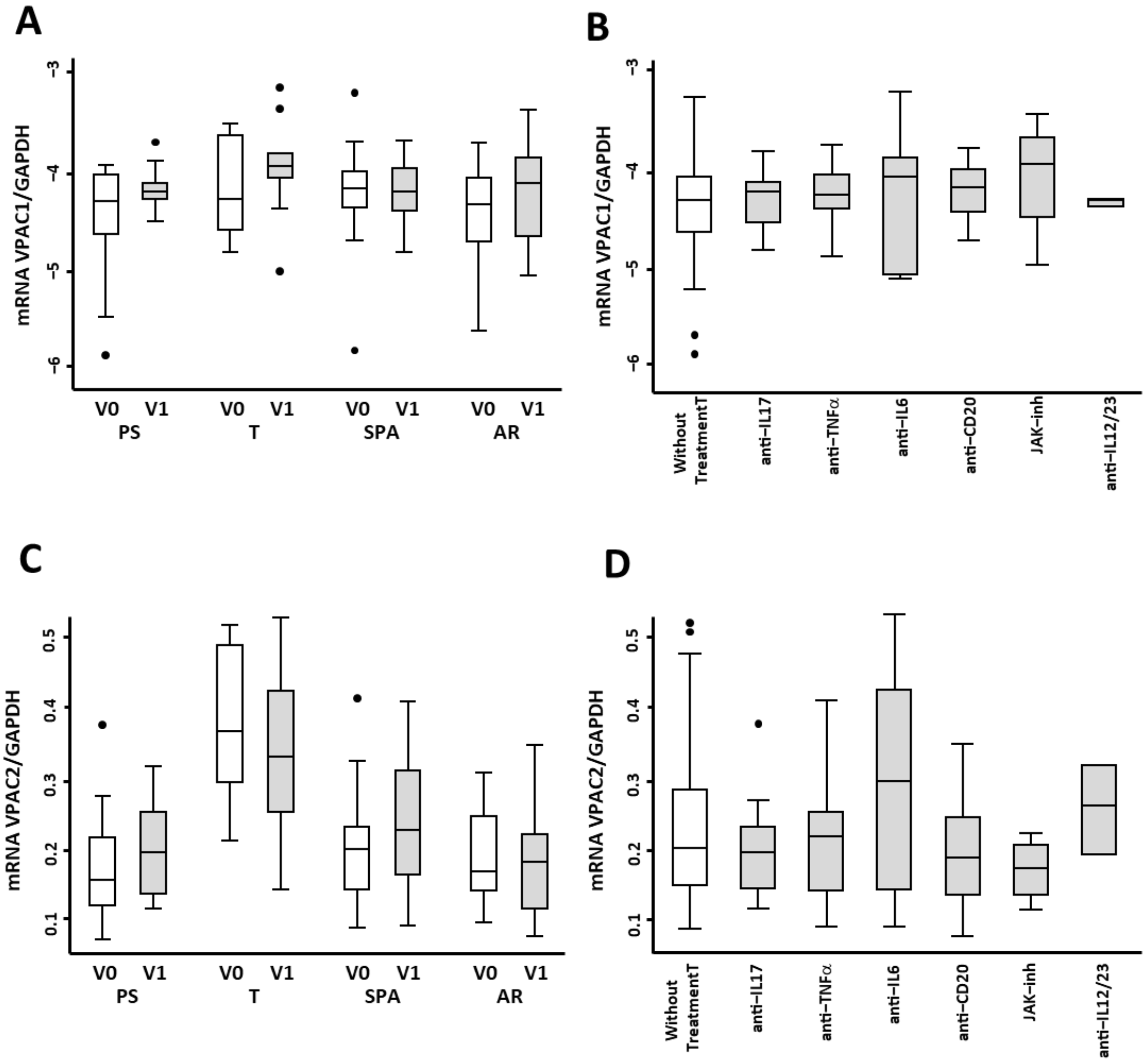

(A) Relative expression of VPAC1/GAPDH mRNA of 15 patients with psoriasis (PS), 8 with Graves´ disease (GD), 15 with spondyloarthritis (SPA) and 14 with rheumatoid arthritis (RA) before (V0) and after 2-4 months (V1) of treatment with biological therapies are shown. **(B)** mRNA relative expression of VPAC1/GAPDH of 38 healthy donors without treatment and 9 IMID patients with anti-IL17 treatment, 15 with with anti-TNFα treatment, 10 with anti-IL6 treatment, 8 with anti-CD20 treatment, 4 with JAK inhibitors and 2 with anti-IL12/23 treatment are shown. **(C)** mRNA of VPAC2/GAPDH of 15 patients with PS, 8 with GD, 15 with SPA and 14 with RA before (V0) and after 2-4 months (V1) of treatment with biological therapies are shown. **(D)** Relative expression of VPAC2/GAPDH mRNA of 38 healthy donors without treatment and 9 IMID patients with anti-IL17 treatment, 15 with with anti-

TNFα treatment, 10 with anti-IL6 treatment, 8 with anti-CD20 treatment, 4 with JAK inhibitors and 2 with anti-IL12/23 treatment are shown.

In panels (A) and (C) the differences in serum VIP levels before and after treatment was analized by paired sign test. Significant differences between groups are shown in each panel. In panels (B) and (D) statistical significance was calculated using ANOVA and Bonferroni correction for multiple comparisons, obtaining the p-values as indicated. Panels data are presented as the interquartile range (p75 upper edge of the box, p25 bottom edge, and p50 midline), p90 and p10 (lines below and above the box) of the serum VIP levels. Dots represent outliers. Significance threshold was set at $p < 0.05$.
